# Outcomes of Pars Plana Vitrectomy in Complicated Retinal Detachment Secondary to Retinal Capillary Hemangioblastoma

**DOI:** 10.3390/medicina61091556

**Published:** 2025-08-29

**Authors:** Pietro Maria Talli, Ginevra Giovanna Adamo, Chiara Vivarelli, Francesco Nasini, Marco Pellegrini, Francesco Parmeggiani, Hassan Al-Dhibi, Sulaiman Alsulaiman, Abdulrahman H. Badawi, Ramzi Judaibi, Paola Ferri, Marco Mura

**Affiliations:** 1 Ophthalmology Clinic Operating Unit, Head and Neck Department, Sant’Anna University Hospital, 44124 Ferrara, Italy; pietro.talli@ospfe.it (P.M.T.); dmagvr@unife.it (G.G.A.); vvrchr@unife.it (C.V.); francesco.nasini@ospfe.it (F.N.); marco.pellegrini@unife.it (M.P.); prmfnc@unife.it (F.P.); 2 Department of Translational Medicine, University of Ferrara, 44121 Ferrara, Italy; 3 Department of Ophthalmology, Ospedali Privati Forlì “Villa Igea”, 47122 Forlì, Italy; 4 Department of Vitreoretinal and Uveitis Divisions, King Khaled Eye Specialist Hospital, Riyadh 11462, Saudi Arabia; hdhibi@kkesh.med.sa (H.A.-D.); ssulaiman@kkesh.med.sa (S.A.); abadawi@kkesh.med.sa (A.H.B.); rjudaibi@kkesh.med.sa (R.J.); 5 Department of Biomedical Metabolic and Neural Sciences, University of Modena and Reggio Emilia, 41121 Modena, Italy; paola.ferri@unimore.it

**Keywords:** endoresection, feeder vessel ligation, retinal capillary hemangioblastoma, Von Hippel–Lindau, vitrectomy, retinal detachment

## Abstract

*Background and Objectives*: Here, we report the anatomical and functional outcomes of Pars Plana Vitrectomy (PPV) with feeder vessel ligation, with or without endoresection in cases of retinal detachment (RD) secondary to retinal capillary hemangioblastoma (RCH). *Materials and Methods*: This retrospective observational study included 12 eyes with RD secondary to RCH. Based on the location of the lesion and the features of the RD, eyes were divided into two groups. Seven eyes with RCH located in Zone 2 or Zone 3, associated with tractional retinal detachment (TRD), underwent PPV with feeder vessel ligation and tumor endoresection. Five eyes, either with RCH in Zone 2 or Zone 3 associated with exudative retinal detachment or with RCH in Zone 1 associated with RD, underwent PPV with feeder vessel ligation alone, without tumor endoresection. Outcome measures included local tumor control, best-corrected visual acuity (BCVA), anatomical success, and rates of complications. *Results*: RCH regressed completely in 100% of eyes with no evidence of recurrence. The mean follow-up was 4.6 years. In the endoresection group, the mean BCVA was 2.18 ± 0.3 logMAR at baseline and 0.95 ± 0.5 logMAR after surgery (*p* = 0.018), whereas in the second group, the baseline mean BCVA was 1.33 ± 0.2 logMAR and 1.52 ± 0.7 logMAR postoperatively. In the first group, retinal attachment was achieved in all eyes, whereas in the second group, two eyes presented with persistent RD and proliferative vitreoretinopathy (PVR). No cases of phthisis bulbi or neovascular glaucoma were observed. *Conclusions*: PPV combined with feeder vessel ligation and endoresection appears to be an effective treatment for TRD secondary to RCH located in Zones 2 and 3, providing satisfactory anatomical and visual outcomes considering the severity of the disease. In cases where tumor location precludes endoresection, PPV with feeder vessel ligation alone may still be a viable option, although the potential risk of PVR could persist.

## 1. Introduction

Retinal capillary hemangioblastoma (RCH) is a rare, benign vascular tumor, often associated with Von Hippel–Lindau (VHL) syndrome. It is characterized by abnormal, dilated, and tortuous feeding and draining blood vessels [1]. RCH can arise near the optic disk (juxtapapillary or epipapillary RCH) or elsewhere within the retina (extrapapillary RCH). The clinical spectrum of RCH varies widely depending on the size and location of the tumor, and it can lead to significant complication, including retinal detachment (RD). The mechanism underlying RD is complex, involving the formation of subretinal fluid due to the leakage from abnormal vessels, tractions caused by the tumor, or retinal ischemia [2,3]. Various treatment modalities for the management of RCH, such as laser photocoagulation, cryotherapy, brachytherapy, and photodynamic therapy, have been described in the literature. Specifically, laser photocoagulation is suggested for extrapapillary RCH up to 1.5 mm, while cryotherapy is commonly used for lesions measuring between 1.5 and 4.5 mm [3]. Additionally, favorable outcomes have been reported with brachytherapy and photodynamic therapy in localized lesions [3]. When RCH is associated with RD, surgical intervention becomes necessary. Although various surgical techniques have been used to manage the detachment and minimize tumor recurrence, standardized treatment guidelines are still lacking [3]. To date, multiple studies have reported suboptimal anatomical and visual outcomes, with high recurrence rates often requiring additional treatments [4,5,6,7]. This study evaluates the anatomical and functional results of Pars Plana Vitrectomy (PPV) combined with feeder vessel ligation, with or without endoresection of the RCH.

## 2. Materials and Methods

### 2.1. Study Design, Setting, and Sample

**Figure 1:**
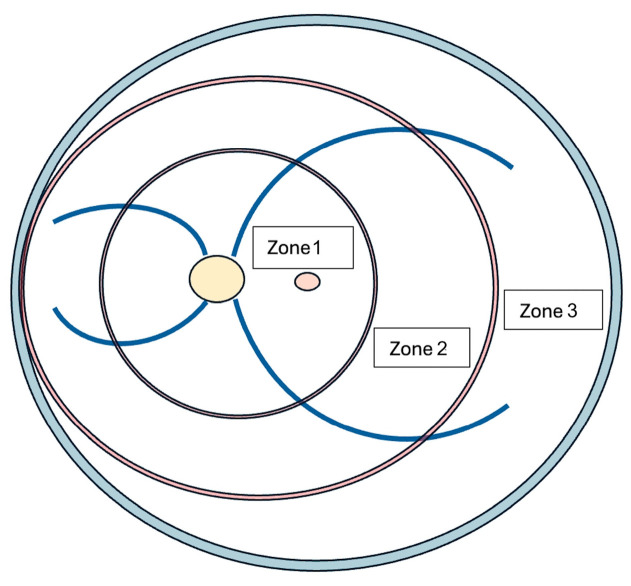
Schematic classification of retinal zones. Zone 1 represents the most central area. Zone 2 is defined as the region surrounding Zone 1, extending to the nasal ora serrata. Zone 3 extends from the outer edge of Zone 2 to the peripheral temporal retina.

This was a retrospective study conducted at King Khaled Eye Specialist Hospital, Riyadh, Saudi Arabia. Here, 12 consecutive eyes of 12 patients with RCH complicated by RD who underwent PPV between January 2014 and December 2022 were enrolled. Genetic testing confirmed VHL syndrome in six patients prior to their referral for ophthalmologic assessment. No specific mutations were identified in the remaining patients. In bilateral cases, the eye affected by RD was included in the analysis, whereas the RCH in the fellow eye, being small in size, was treated with laser photocoagulation. The surgical approach was determined based on the location of the RCH and the characteristics of the associated RD. For this purpose, the retina was conceptually divided into three zones, and the treatment strategy was tailored according to the zone in which the RCH was located (Figure 1).

Zone 1 was defined as the most central area, comprising a circle centered on the optic disk with a radius equal to twice the distance between the optic disk and the macula. Zone 2 was defined as the area surrounding Zone 1, extending from its outer edge to the nasal ora serrata. Zone 3 included the remaining temporal crescent of the retina, extending from the outer edge of Zone 2 to the peripheral retina. Based on this classification, patients with RCH located in Zone 2 or Zone 3 associated with tractional retinal detachment (TRD) underwent PPV, feeder vessel ligation, and retinectomy with tumor endoresection. Patients with RCH located in Zone 1 associated with RD, as well as those with RCH in Zone 2 or 3 with exudative retinal detachment (ERD), underwent PPV and feeder vessel ligation without tumor endoresection. In borderline or uncertain cases, intraoperative evaluation, by testing retinal rigidity and observing the effects of perfluorocarbon liquid following a peripheral retinotomy, can help guide surgical decision making.

### 2.2. Eye Examination

All patients underwent a comprehensive ophthalmological examination before surgery and postoperatively. The examination included best-corrected visual acuity (BCVA), slit-lamp biomicroscopy, fundoscopy, spectral-domain optical coherence tomography (SD-OCT) (Heidelberg Engineering, Heidelberg, Germany), ultra-widefield fundus photography, and fluorescein angiography (Optos PLC, Dunfermline, UK).

### 2.3. Surgical Technique

All cases underwent chandelier-assisted 23-gauge PPV under either local sub-Tenon or general anesthesia. Following core vitrectomy, a posterior vitreous detachment was induced by staining the vitreous with triamcinolone acetonide. A complete vitrectomy with vitreous base shaving was performed, and all preretinal membranes were dissected using a bimanual approach.

In the resection group, perfluorocarbon liquid was injected into the vitreous cavity to stabilize the retina. One trocar was then removed, and a single-armed 10-0 Prolene suture was inserted through the sclerotomy and tied bimanually around the feeder vessel using two intraocular serrated-jaw forceps. Endodiathermy was performed around the tumor lesion. Retinectomy and endoresection of the tumor were carried out using the vitrectomy probe after increasing the intraocular pressure to 60 mmHg to reduce the risk of bleeding. Subretinal exudates were aspirated when present. Silicone oil was used as an endotamponade in all patients following direct perfluorocarbon–silicone oil exchange or fluid–air exchange. Endolaser retinopexy was performed at the edges of the retinectomy and around any retinal breaks. The surgical procedure is demonstrated in Supplemental Video S1.

In the ligation group, endoresection was not performed; instead, feeder vessels were ligated with 10-0 Prolene to isolate the lesions from the retinal vascular circulation. Fluid–air exchange was carried out following the retinotomy. Silicone oil was used as an endotamponade in four eyes and SF_6_ 20% gas in one eye.

### 2.4. Ethical Considerations

This study was conducted in accordance with the Declaration of Helsinki and was approved by the Institutional Review Board of King Khaled Eye Specialist Hospital (RD/26001/IRB/0340-25; 8 April 2025).

### 2.5. Statistical Analysis

Data analysis was performed using SPSS (version 29.0, IBM Corp., Armonk, NY, USA). Continuous variables are expressed as mean ± standard deviation and median, while categorical variables are presented as counts and percentages. The Shapiro–Wilk test revealed that the data were not normally distributed; therefore, the Wilcoxon signed-rank test was used to compare preoperative and postoperative BCVA. A *p*-value less than 0.05 was considered statistically significant.

## 3. Results

The study cohort consisted of 12 eyes from 12 patients (7 males and 5 females) with a mean age of 20.7 ± 9.5 years (Table 1). Six of the patients had von Hippel–Lindau syndrome. In eleven cases, the tumor was in the peripheral retina, whereas in one case, the tumor was located above the optic disk. The patient with epipapillary RCH had another RCH in the peripheral retina of the same eye. The sizes of the RCHs ranged from 3 to 12 disk diameters (DD) (two were 5 DD, one was 6 DD, one was 7 DD, two were 9 DD, four were 10 DD, and one was 12 DD). The epipapillary RCH measured 3 DD. Bilateral involvement was observed in 30% of cases. Subretinal hard exudate accumulation was present in seven eyes.

Seven eyes from seven patients (four males and three females) with a mean age of 18.1 ± 8.6 years underwent ligation with endoresection (Table 2). Five eyes from five patients (three males and two females) with a mean age of 24.2 ± 10.6 years underwent ligation without endoresection. There were no intraoperative complications. In six eyes, subretinal exudate in the macular area was aspirated using a vitrector through a retinotomy.

In the whole sample, an average of 2.17 ± 0.84 surgeries were performed. In the endoresection group, which included seven eyes, an average of 2.14 ± 0.38 surgeries were performed. In the ligation-only group, consisting of five eyes, an average of 2.20 ± 1.30 surgeries were performed (Table 3).

**Table 2:** Baseline BCVA and intraoperative procedures of the patients who underwent PPV for complex RD secondary to RCH.

Case (n)	Pre-BCVA (Snellen)	Pre-BCVA (LogMAR)	Initial Surgery	Endoresection	Ligation
1	5/200	1.6	PPV, EL, SOI	Yes	Yes
2	3/300	2.17	PPV, EL, SOI	Yes	Yes
3	HM	2.3	PPV, EL, SOI	Yes	Yes
4	HM	2.3	PPV, EL, SOI	Yes	Yes
5	HM	2.3	PPV, EL, SOI	Yes	Yes
6	HM	2.3	PPV, EL, SOI	Yes	Yes
7	HM	2.3	PPV, EL, SOI	Yes	Yes
8	20/400	1.3	PPV, EL, gas	No	Yes
9	4/200	1.69	PPV, SOI	No	Yes
10	20/300	1.17	PPV, EL, Cryo, SOI	No	Yes
11	20/400	1.3	PPV, EL, Cryo, SOI	No	Yes
12	20/300	1.17	PPV, EL, Cryo, SOI	No	Yes

EL = endolaser; SOI = silicone oil injection; Cryo = cryotherapy; HM = hand motion.

**Table 3:** Intraoperative procedures and postoperative anatomical and visual outcomes of the patients who underwent PPV for complex RD secondary to RCH.

Case (n)	Additional Surgery	Surgeries (n)	Follow-Up (years)	Final Retinal Status	Post-BCVA (Snellen)	Post-BCVA (LogMAR)
1	Phaco, SO removal, MP	2	5	Attached	20/200	1
2	Phaco, SO removal	2	8	Attached	20/70	0.54
3	PPV, MP, SO removal	3	3	Attached	20/200	1
4	SO removal	2	3	Attached	2/200	2
5	SO removal, MP	2	5	Attached	20/100	0.69
6	SO removal, MP	2	4	Attached	20/160	0.9
7	SO removal	2	5	Attached	20/70	0.54
8	None	1	4	Attached	20/200	1
9	None	1	5	Attached	20/200	1
10	SO removal	2	2	Attached	20/200	1
11	PPV, MP	4	4	Detached with PVR	HM	2.3
12	PPV, MP	3	7	Detached with PVR	HM	2.3

Phaco = lens phacoemulsification; SO removal = silicone oil removal; MP = membrane peeling.

### 3.1. Anatomical Outcomes

The mean follow-up period was 4.7 ± 1.7 years in the endoresection group and 4.4 ± 1.8 years in the ligation group. RCHs regressed completely in 100% of eyes, with no evidence of recurrence.

**Figure 2:**
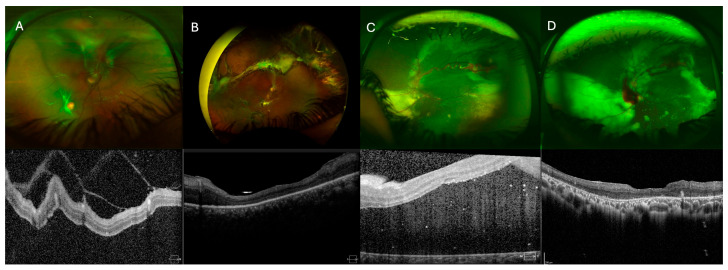
(**A**,**B**) Ultra-widefield fundus photographs and macular SD-OCT images before and 1 year after surgery (PPV + feeder vessel ligation with tumor resection). Images show the retina of a 16-year-old female patient’s right eye with a superior RCH and a small RCH in the inferior retina. TRD and ERD are secondary to the superior RCH. SD-OCT images show macular area before and after surgery. (**C**,**D**) Ultra-widefield fundus photographs and macular SD-OCT images before and 1 year after surgery (PPV + feeder vessel ligation with tumor resection). Images show the retina of a 12-year-old male patient’s left eye with a superotemporal RCH and secondary TRD and ERD. SD-OCT images show the macular area before and after surgery.

**Figure 3:**
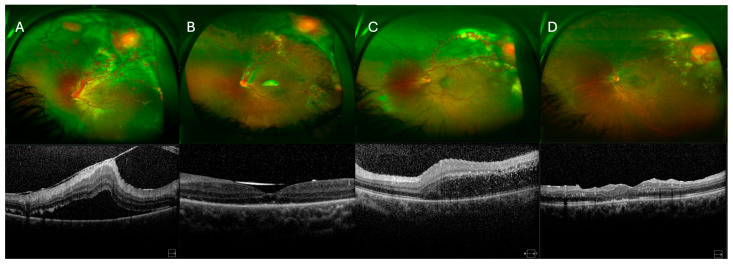
(**A**,**B**) Ultra-widefield fundus photographs and macular SD-OCT images before and 1 year after surgery (PPV + feeder vessel ligation without tumor resection). Images show the retina of a 14-year-old male patient’s left eye with two superior RCHs and ERD. SD-OCT images show macular area before and after surgery. (**C**,**D**) Ultra-widefield fundus photographs and macular SD-OCT images before and 1 year after surgery (PPV + feeder vessel ligation without tumor resection). Images show the retina of a 21-year-old female patient’s left eye with a superotemporal RCH and ERD. SD-OCT images show the macular area before and after surgery.

Retinal attachment was achieved in 10 eyes (83.3%) (Table 3). In the endoresection group, retinal reattachment was achieved in all eyes following silicone oil removal. In contrast, at the end of the follow-up period in the ligation-only group, two eyes presented an attached retina, one eye had an attached retina under silicone oil (which was not removed), and two eyes, despite multiple surgeries, had a persistently detached retina with fibrosis under silicone oil. Representative cases from the endoresection and ligation-only groups, including pre- and postoperative ultra-widefield fundus photographs and SD-OCT images, are shown in Figure 2 and Figure 3, respectively, illustrating the anatomical outcomes following different surgical approaches.

In the ligation without endoresection group, proliferative vitreoretinopathy (PVR) developed in two of five eyes, resulting in recurrent RD as a short-term complication. Silicone oil-associated glaucoma, which required antiglaucoma medications, developed in one eye (8.3%) in the endoresection group. Additionally, four of the seven eyes in the endoresection group developed an epiretinal membrane, and macular peeling was performed during the second surgery.

Cataract surgery was performed in two eyes during the follow-up period. No cases of phthisis bulbi or neovascular glaucoma were registered in our cohort of patients. No enucleation was necessary over the follow-up period. No suture complications, such as hemorrhage or reproliferation from the ligature, were observed during the follow-up. There were no tears at the vessel ligature.

### 3.2. Functional Outcomes

The mean preoperative BCVA was 1.83 ± 0.5 logarithm of the minimum angle of resolution (logMAR) (median = 1.93), which improved to 1.19 ± 0.6 logMAR (median = 1.00) postoperatively (*p* = 0.041) (Table 2 and Table 3). At the end of the follow-up period, BCVA improved in 10 eyes and worsened in 2 eyes. In the endoresection group, the mean BCVA was 2.18 ± 0.3 logMAR (median = 2.30) at presentation and improved to 0.95 ± 0.5 logMAR (median = 0.90) after surgery (*p* = 0.018). In contrast, in the ligation-only group, the mean BCVA was 1.33 ± 0.2 logMAR (median = 1.30) preoperatively and 1.52 ± 0.7 logMAR (median = 1.00) postoperatively, with no statistically significant difference (*p* = 0.69).

## 4. Discussion

RCH associated with RD is a rare and challenging condition. Timely surgical intervention is critical to prevent complications such as permanent vision loss, neovascular glaucoma, and phthisis bulbi [5,8,9]. However, due to the rarity of this condition, there is still no consensus on the optimal surgical approach, and to our knowledge, many studies in the literature report suboptimal visual and anatomical outcomes, with high recurrence rates [4,5,6,7,9]. In 2001, Farah et al. proposed transretinal feeder vessel ligation, which resulted in a reduction in RCH size, although two new feeder vessels developed [10]. Subsequently, Khurshid et al. recommended combining feeder vessel ligation with tumor endoresection and laser treatment around the tumor site to mitigate the risks of postoperative hemorrhage during the involution of feeder vessels [11]. Conversely, in 2017, Avci et al. proposed combined PPV, endodiathermy cauterization of feeder vessels followed by endoresection of the lesion through retinectomy [6]. They reported favorable anatomical and functional outcomes; however, some patients experienced recurrence of RCH and development of PVR. In our study, surgical management was tailored according to the anatomical location of the tumor and the characteristics of the associated RD.

### 4.1. Anatomical Outcomes

In our study, complete tumor control was achieved in both the endoresection group and the group treated with feeder vessel ligation alone, with no evidence of recurrence observed during the follow-up period. The absence of recurrences may be partially attributed to the relatively low proportion of patients with VHL disease in our cohort, as these individuals are at higher risk for multifocal and recurrent lesions due to their underlying genetic predisposition.

The endoresection group demonstrated superior anatomical results, with retinal reattachment maintained in all cases at the end of follow-up. In contrast, retinal attachment was achieved in only three of five eyes in the ligation-only group. This disparity may reflect a higher risk of PVR and redetachment in the ligation group, potentially due to incomplete tumor devascularization and subsequent reperfusion through collateral or newly formed feeder vessels.

Our findings are consistent with those of van Overdam et al., who suggested that complete tumor excision reduces the risk of PVR by enabling removal of the adherent epiretinal membrane surrounding the lesion [9]. Although Gaudric et al. reported an increased rate of PVR in eyes with retinectomy, they proposed that the higher complication rate and poor outcomes in the retinectomy group may be due to more severe initial presentations [4].

Indeed, endoresection carries several potential complications, including intraoperative hemorrhage, iatrogenic retinal breaks, and postoperative PVR [11,12]. These risks are particularly pronounced in younger patients, in whom the posterior hyaloid is often tightly adherent to the retina, increasing the likelihood of mechanical trauma during posterior vitreous detachment induction. Moreover, the creation of a retinotomy or retinectomy may facilitate the dispersion of retinal pigment epithelial cells into the vitreous cavity, a well-documented trigger for PVR. Additionally, surgical manipulation of the tumor may transiently elevate intraocular inflammatory markers, further promoting fibrotic membrane formation.

A theoretical concern with endoresection is that transecting the tumor with a vitreous cutter could lead to the dissemination of interstitial tumor cells, potentially seeding recurrence [7]. However, in sporadic RCH, this risk appears low, and no recurrences were observed in our endoresection group. In VHL patients, new or recurrent lesions are more likely due to the systemic nature of the disease rather than surgical dispersion.

Alternative approaches such as endodiathermy cauterization of feeder vessels followed by tumor aspiration also carry substantial risks. These include intraoperative and postoperative vitreous hemorrhage, which may exacerbate inflammation and promote PVR [13,14,15]. Extensive diathermy can disrupt the blood–ocular barrier, leading to chronic low-grade inflammation and epiretinal membrane formation [11,16]. Furthermore, laser photocoagulation, though less invasive, often fails in thicker tumors, resulting in incomplete obliteration, reperfusion, and persistent or recurrent ERD [4]. The relatively high recurrence rates after laser or diathermy monotherapy underscore the limitations of non-excisional strategies in achieving durable anatomical control.

Notably, advances in vitreoretinal surgery since the late 1990s have reduced surgical trauma and enhanced fluidics. These innovations may partially explain the improved outcomes in our endoresection group compared to earlier series like Gaudric et al., where larger-gauge instruments and less refined techniques were used [4].

In our study, in the group that underwent endoresection of the tumor, four of seven eyes developed an epiretinal membrane, and macular peeling was performed during the second surgery without complications. Epiretinal membrane formation is influenced by several factors related both to the underlying disease and to surgical interventions such as laser photocoagulation, cryotherapy, retinotomy, and retinectomy. Although significant, this complication is acceptable considering the natural history of the disease.

Silicone oil was used as a long-acting tamponade in almost all cases and was removed between 6 and 12 months postoperatively, once retinal stability was confirmed, and no signs of recurrent traction or exudation were present. In complex RCH-associated detachments, especially in VHL patients, silicone oil provides a more durable tamponade than gas, reducing the risk of redetachment. However, in eyes with poor visual potential and high PVR risk, permanent oil retention may be a reasonable option to preserve anatomical integrity, despite long-term complications such as glaucoma, keratopathy, or cataract.

Importantly, no cases of phthisis bulbi or neovascular glaucoma were observed in our cohort, in contrast to prior reports [5,9]. This favorable outcome underscores the importance of early surgical intervention and meticulous management of vascular and inflammatory complications.

### 4.2. Functional Outcomes

In our series, BCVA overall improved in 83.3% of cases. Specifically, in the endoresection group, we observed a significant improvement in final BCVA, although eyes were more severely affected at baseline. In the ligation-only group BCVA improved in three of five patients, whereas two patients experienced persistent RD under silicone oil due to fibrosis, contributing to poorer outcomes.

These findings contrast with prior studies by Gaudric et al. and Krzystolik et al., which reported worse visual outcomes in cases treated with retinectomy and endoresection compared to those treated with laser. However, the poorer outcomes in their series may reflect the advanced disease and poor baseline BCVA in these cases [4,5].

A direct comparison with the study by Raval et al. is challenging, as they categorized their sample based on tumor size, whereas we based our treatment decisions on the features of RD and the location of the tumor [17]. In Raval et al.’s series, tumors smaller than 2.5 mm were treated with PPV and laser or cryotherapy [17], whereas when the tumor size was more than 2.5 mm, PPV with endoresection of the tumor was carried out in combination with cauterization, or ligation of feeder vessels was performed.

### 4.3. Surgical Techniques and Considerations

Complete removal of subretinal exudates was a key step in improving visual outcomes, particularly in macular-involving cases. We also found internal limiting membrane (ILM) peeling to be a valuable adjunct in reducing the risk of macular pucker, in line with van Overdam et al. [9]. However, given the young age of the patients, we often deferred ILM peeling to a secondary procedure, performing it only when epiretinal membrane formation was evident. Mallmann et al. described a “double peeling” technique, removing both the epiretinal membrane and ILM, in VHL-related RD, which may offer additional protection against recurrence of macular pathology [13]. We support this approach in selected cases with high PVR risk. To minimize PVR, we advocate for a complete vitrectomy, including meticulous removal of the posterior hyaloid after triamcinolone-assisted staining, as suggested by van Overdam et al. and Huang et al. [9,12]. The residual hyaloid cortex can serve as a scaffold for glial and fibrovascular proliferation, contributing to tractional redetachment. Early surgical intervention, before the development of extensive fibrosis, is also critical to preserving visual potential.

The management of juxtapapillary RCH remains particularly challenging. Thermal laser carries a high risk of optic nerve damage, leading to altitudinal defects or central scotomas [3]. While photodynamic therapy, intravitreal anti-Vascular Endothelial Growth Factor agents (anti-VEGF), and corticosteroids have been used in selected cases, they are often insufficient when extensive RD is present. In such scenarios, PPV with feeder vessel ligation remains the only viable option to achieve anatomical reattachment and prevent further vision loss.

### 4.4. Limitations and Future Directions

Our study has several limitations. Its retrospective design and small sample size restrict the power and generalizability of our findings. Additionally, variations in lesion size, features of RD, and the presence or absence of underlying VHL disease make comparisons with other studies challenging. Future prospective studies with larger cohorts and longer follow-up are needed to validate these results. Furthermore, incorporating supplementary functional testing, such as contrast sensitivity, visual field assessment, and electroretinography, may provide a more comprehensive understanding of visual outcomes and retinal function following surgery.

## 5. Conclusions

Patients with RD secondary to RCH may preserve both anatomical integrity and visual function when managed with timely surgical intervention. In our series, PPV combined with feeder vessel ligation and tumor endoresection was associated with favorable anatomical and functional outcomes in complex cases involving both exudative and tractional components.

When feasible, combining feeder vessel ligation with endoresection may offer advantages in reducing the risk of tumor reperfusion and recurrence, particularly in larger or more vascular lesions. However, this approach requires careful surgical technique and carries a non-negligible risk of complications, including epiretinal membrane formation and iatrogenic retinal breaks.

In cases where endoresection is not feasible or deemed too high risk, such as in juxtapapillary tumors, feeder vessel ligation alone may be considered, but these patients require close and long-term follow-up to monitor for signs of tumor recurrence or proliferative vitreoretinopathy.

Given the rarity of the condition and the heterogeneity of presentation, treatment should be individualized based on tumor location, extent of RD, and patient-specific factors. Further multicenter studies with larger cohorts and longer follow-up are needed to better define the optimal surgical strategy for this challenging condition.

## Figures and Tables

**Table 1:** Demographic characteristics and preoperative anatomical findings of the patients undergoing PPV for complex RD secondary to RCH.

Case (n)	Age (years)	Gender	Eye	Tumor Dimension (DD)	Subretinal Macular Exudate	Initial Retinal Status
1	12	Male	Left	5	Yes	ERD and TRD
2	37	Male	Right	5	Yes	TRD
3	16	Female	Right	10	No	TRD
4	16	Female	Right	9	Yes	ERD
5	13	Male	Right	10	Yes	TRD
6	19	Female	Left	9	Yes	TRD
7	14	Male	Right	12	Yes	TRD
8	14	Male	Left	10	Yes	ERD
9	21	Female	Left	6	No	TRD
10	42	Male	Left	3	No	ERD
11	24	Male	Left	7	No	ERD
12	20	Female	Right	10	No	ERD

## Data Availability

The original contributions presented in this study are included in the article/supplementary material. Further inquiries can be directed to the corresponding author.

## Supplementary Materials

The following supporting information can be downloaded at: https://www.mdpi.com/article/10.3390/medicina61091556/s1, Video S1: The surgical procedure.

## Abbreviations

The following abbreviations are used in this manuscript:

RD	Retinal Detachment
RCH	Retinal Capillary Hemangioblastoma
BCVA	Best-Corrected Visual Acuity
VHL	Von Hippel–Lindau
PPV	Pars Plana Vitrectomy
SD-OCT	Spectral-Domain Optical Coherence Tomography
DD	Disk Diameters
PVR	Proliferative Vitreoretinopathy
TRD	Tractional Retinal Detachment
ERD	Exudative Retinal Detachment
Anti-VEGF	Anti-Vascular Endothelial Growth Factor
Phaco	Phacoemulsification
Cryo	Cryotherapy
SO	Silicone Oil
SOI	Silicone Oil Injection
MP	Membrane Peeling
EL	Endolaser
HM	Hand Motion
LogMAR	Logarithm of the Minimum Angle of Resolution
ILM	Internal Limiting Membrane
